# Methylation analysis by targeted bisulfite sequencing in large for gestational age (LGA) newborns: the LARGAN cohort

**DOI:** 10.1186/s13148-023-01612-8

**Published:** 2023-12-13

**Authors:** Tamara Carrizosa-Molina, Natalia Casillas-Díaz, Iris Pérez-Nadador, Claudia Vales-Villamarín, Miguel Ángel López-Martínez, Rosa Riveiro-Álvarez, Larry Wilhelm, Rita Cervera-Juanes, Carmen Garcés, Alejandro Lomniczi, Leandro Soriano-Guillén

**Affiliations:** 1 Department of Pediatrics, IIS-Fundación Jiménez Díaz, Universidad Autónoma de Madrid, Avda. Reyes Católicos, 2, 28040 Madrid, Spain; 2 Lipid Research Laboratory, IIS-Fundación Jiménez Díaz, Madrid, Spain; 3 Department of Genetics and Genomics, IIS-Fundación Jiménez Díaz, Universidad Autónoma de Madrid, Madrid, Spain; 4 Department of Physiology and Pharmacology, Center for Precision Medicine, Wake Forest University School of Medicine, Winston-Salem, NC USA; 5 Department of Physiology and Biophysics, Dalhousie University School of Medicine, 5850 College Street, Halifax, NS B3H 4R2 Canada

**Keywords:** Epigenome, Markers, Neonatal, Cardiovascular, Kidney, Development

## Abstract

**Background:**

In 1990, David Barker proposed that prenatal nutrition is directly linked to adult cardiovascular disease. Since then, the relationship between adult cardiovascular risk, metabolic syndrome and birth weight has been widely documented. Here, we used the TruSeq Methyl Capture EPIC platform to compare the methylation patterns in cord blood from large for gestational age (LGA) vs adequate for gestational age (AGA) newborns from the LARGAN cohort.

**Results:**

We found 1672 differentially methylated CpGs (DMCs) with a nominal *p* < 0.05 and 48 differentially methylated regions (DMRs) with a corrected *p* < 0.05 between the LGA and AGA groups. A systems biology approach identified several biological processes significantly enriched with genes in association with DMCs with FDR < 0.05, including regulation of transcription, regulation of epinephrine secretion, norepinephrine biosynthesis, receptor transactivation, forebrain regionalization and several terms related to kidney and cardiovascular development. Gene ontology analysis of the genes in association with the 48 DMRs identified several significantly enriched biological processes related to kidney development, including mesonephric duct development and nephron tubule development. Furthermore, our dataset identified several DNA methylation markers enriched in gene networks involved in biological pathways and rare diseases of the cardiovascular system, kidneys, and metabolism.

**Conclusions:**

Our study identified several DMCs/DMRs in association with fetal overgrowth. The use of cord blood as a material for the identification of DNA methylation biomarkers gives us the possibility to perform follow-up studies on the same patients as they grow. These studies will not only help us understand how the methylome responds to continuum postnatal growth but also link early alterations of the DNA methylome with later clinical markers of growth and metabolic fitness.

**Supplementary Information:**

The online version contains supplementary material available at 10.1186/s13148-023-01612-8.

## Introduction

The term large for gestational age (LGA) newborn is defined as a newborn with a gestational age- and gender-specific weight and/or length higher than + 2 SDS [1]. The identified factors related to LGA newborn etiology could be grouped into fetal, maternal and uteroplacental factors [2]. Among maternal factors, it is worth noting the relevance of the association between maternal gestational diabetes and overgrowth due to continuous stimulus of high glucose levels that leads to endogenous fetal overproduction of insulin-like growth factor-1 (IGF-1) and insulin, which, as a result, induces macrosomia [3]. Notably, fetal growth is closely related to maternal body size and maternal health [1]. However, in some cases, it is not possible to determine the exact mechanism causing fetal growth disturbances.

In 1990, David Barker proposed that prenatal nutrition is directly linked to adult cardiovascular disease, in what is now known as the fetal origin of adult disease hypothesis [4, 5]. Since then, the relationship between birth weight, adult cardiovascular risk, metabolic syndrome, and type 2 diabetes has been widely documented [6–8]. This association is not only described with high birth weight but also with low birth weight, establishing a U-shaped cardiometabolic risk link [1]. Nevertheless, today, we cannot explain this association after a notably long period of latency [9]. Recently, researchers have focused on epigenetic modifications as a possible mechanism involved in how alterations during the fetal period affect overall adult health and disease risk [10].

Epigenetics is the study of reversible and heritable changes in gene expression without changes in DNA sequence [11]. This puts epigenetic mechanisms at the center of environmental–gene interactions, where changes in the environment influence the epigenetic landscape at gene regulatory regions, which could ultimately contribute to variations in gene expression, alterations in fetal growth [12] and/or cardiometabolic function [13]. To date, there are different ways to evaluate DNA methylation [14]. Whole-genome bisulfite sequencing (WGBS) offers the best genomic coverage for DNA methylation evaluation, assuming a very elevated budget and a large amount of data that makes its interpretation extremely hard. For these reasons, new platforms such as the Methylation EPIC Beadchip Microarray (EPIC-array) and TruSeq Methyl Capture EPIC (TruSeq EPIC) have emerged [15, 16]. TruSeq EPIC includes new epigenetic areas of interest compared to EPIC-array and uses next generation sequencing to pull off targeted bisulfite sequencing covering 3.34 million CpG sites.

In recent years, several studies have demonstrated a relationship between newborn body size and DNA methylation patterns in genes related to fetal growth, metabolism and cardiometabolic health [17–21]. Most of these studies used Illumina the 27 K, 450 K or Infinium 1.0 Bead Array (850 K CpGs) to study methylation changes in placental tissue and are focused on the effect of intrauterine growth restriction. Previous studies used cord blood or placental tissue to identify methylation markers correlated with birth weight in candidate genes or using Infinium Arrays. This was excellently shown in a recent meta-analysis of epigenome-wide association studies (EWAS) using birthweight as a continuous variable of 8,825 newborns from 24 different cohorts [22].

For the present study, we opted for the TruSeq Methyl Capture EPIC platform because it utilizes target-specific bait sequences covering 3.34 million CpG sites that target regulatory regions such as CpG islands, CpG shores, CpG shelves, TSS200, and promoter regions. This approach presents an attractive cost-effective alternative to uncover novel disease-associated genomic loci in EWAS and overcomes the limitations of lower genome coverage (Infinium 450/800 K) arrays, high cost and processing time (WGBS), while avoiding overrepresentation of repeated (RRBS) and methylated regions (MeDIP-Seq).

Another relevant issue is to select the most appropriate tissue to evaluate DNA methylation that reflects the metabolic milieu of the fetus. In this regard, attending to the main objectives of our study, we had to choose among placenta, umbilical cord tissue and umbilical cord blood. Most studies aimed at identifying epigenetic markers of overgrowth used placental tissue with standardized operating procedures to minimize sampling of the maternal side [20, 23, 24]. On the other hand, in umbilical cord tissue and umbilical cord blood, we can obtain only fetal cells [25]. We decided to perform methylation profiling from umbilical cord blood because its cell type proportion is only dependent on gestational age [25].

In this pilot study, we used the TruSeq Methyl Capture EPIC platform to identify differential methylation patterns in cord blood from a small cohort of LGA and adequate for gestational age (AGA) newborns.

## Materials and methods

### Type of study

Our pilot study was conducted between March 2019 and December 2022 in the Pediatric Department of Fundación Jiménez Díaz Hospital, located in Madrid, Spain.

We used a small sample size of 25 individuals divided into thirteen large for gestational newborns (LGA) and twelve adequate for gestational age newborns (AGA: control group) matched by sex and mode of delivery. These newborns will be followed up until at least pubertal age, establishing the LARGAN (*Large for Gestational Age Newborns*) cohort.

### Subjects

We included LGA and AGA patients who were born at our institution.

### Inclusion criteria

LGA newborns were ≥ 34 weeks of gestational age, whose weight and/or length were >+ 2 SDS (Z-score) according to sex-specific birth weight and birth length gestational age reference charts [26]. AGA newborns were ≥ 34 weeks of gestational age, whose weight and length were between − 2 and + 2SDS according to sex-specific birth weight and birth length gestational age reference charts [26].

### Exclusion criteria

Prenatal and/or postnatal suspicion of any syndrome; any major structural malformation; postnatal suspicion of mild or severe encephalopathy due to infection, hypoxia-ischemia, or metabolic etiology; impossibility of measuring weight/length in the first 24 h of life due to the patient's clinical severity and refusal of parents to be included in the study.

### Ethics approval and consent to participate

The study protocol was approved by the institutional review board of the University Hospital Fundación Jiménez Díaz (code: PIC003-19, approval date: 1/29/2019). Parents signed a written informed consent form after the nature of all procedures had been fully explained at the time of enrollment. The collection of samples belongs to the Biobank of the University Hospital Fundación Jiménez Díaz. This investigation was carried out in adherence to the principles of the Declaration of Helsinki and subsequent reviews, as well as Spanish legislation in force on clinical research in human subjects.

### Data collection

These data were collected from both questionnaires completed by families and from medical records.

### Family data

Mother’s race (attending to our reference population, we have included the following groups: White, Hispanic, Black, Asian and North African). Maternal age at delivery. Mother’s weight, height, and body mass index [BMI: weight (kg)/height^2^ (m)] at the beginning of pregnancy and at delivery.

### Obstetric history

Pregestational comorbidities (chronic hypertension, diabetes), maternal tobacco consumption, results of prenatal ultrasounds, weight gain during pregnancy, appearance of comorbidities during gestation such as hypertension and glucose metabolic disturbances (diabetes or impaired glucose tolerance).

### Newborn data

Gestational age (this variable was determined from the date of the last menstrual period and was confirmed by early ultrasound), type of delivery, Apgar score, gender, weight [grams, SDS 26], length [cm, SDS 26], head circumference [cm, SDS 27], and ponderal index [PI = 100 × weight (grams)/length (cm)^3^]. Weight, length, and head circumference were determined within the first 6 h of life. Weight was measured with a newborn electronic scale; length was determined by an infantometer, and head circumference was determined by a nonelastic tape.

## Sample processing

### DNA extraction

Umbilical cord blood UCB was collected by trained staff at the time of delivery. Genomic DNA was immediately extracted from whole umbilical cord blood using an automated DNA extractor (BioRobot EZ1, QIAGEN, Hilden, Germany).

### Library construction and sequencing

A genome-wide bisulfite sequencing approach was performed to specifically study DNA methylation using the Illumina TruSeq Methyl Capture EPIC kit (Illumina, Cambridge, UK), which targets over 3.3 million CpGs. Libraries were prepared following the manufacturer’s protocol. Briefly, DNA samples were quantified using a fluorometric method (Qubit 3.0 Fluorometer, Life Technologies), diluted to 500 ng of total starting material at 10 ng/µl, and fragmented on an S2 sonicator (Covaris, Woburn, MA, USA), followed by end repair. After adapter ligation, hybridization and capture, libraries were pooled into 4 samples at a time and subjected to bisulfite conversion, PCR amplification and clean-up. Finally, before sending the libraries to the sequencing core, they were checked for integrity and size distribution using a Bioanalyzer High Sensitivity Kit (Agilent, Santa Clara, CA). Finally, pooled libraries were loaded into a NextSeq500 flow cell and sequenced using the NextSeq500 High Output Reagents Kit (Illumina, Cambridge, UK) to obtain 300-bp paired-end reads (with an average 40 × coverage and > 90% of target bases covered at ≥ 10 ×).

### Statistical and bioinformatic analysis

The quality of the bisulfite-converted sequencing reads was assessed with FastQC. Reads were trimmed and aligned to the human reference genome (GRCh38/hg38), and then the bisulfite conversion rates were evaluated, ensuring all libraries were > 98% converted, and CpG methylation was evaluated using Bismark [28]. The methylation rates were calculated as the ratio of methylated reads over the total number of reads. Methylation rates for CpGs with fewer than 5 reads were excluded from further analysis. The RnBeads filtering module was set for SNP filtering, removal of sex chromosome, and removal of high coverage outliers [29]. Filtering for missing quantile values was set to 0.05, and a filtering deviation threshold of 0.005, with no imputation method employed.

Surrogate variable analysis and differential DNA methylation analysis were carried out using the R package RnBeads version 2.0 [29]. To adjust for potential hidden confounders, including cell proportion variability, surrogate variable analysis (SVA) was applied to the AGA-LGA group comparison. The surrogate variables (SVs) that accounted for the unexplained variance not correlated with the variable of interest (“group”) were collected and applied as covariates in the differential methylation analysis. Differential methylation between groups was analyzed using the empirical Bayesian generalized linear model built-in the limma package [30], implemented in the RnBeads package. We included “group” as differential comparison columns and “gest_age,” “gender,” “type_of_delivery” as well as all quantified maternal anthropometric, demographic and comorbidity factors (see Table 2) as covariates in the linear model. We applied correction for multiple comparisons to identify DMCs at FDR < 0.05, identifying only two DMCs with significant FDR; for that, all Gene Enrichment studies were performed with DMCs at nominal (uncorrected) *p* values. In parallel, the DMCs at nominal *p* values from the linear regression analysis were used as input for Comb-p [31] analysis to identify differentially methylated regions (DMRs). Comb-p uses a sliding window correction where each Wilcoxon *P* value is adjusted by applying the Stouffer–Liptak–Kechris (slk) method of neighboring *P* values as weighted according to the observed autocorrelation (ACF) at the appropriate lag [32]. In summary, comb-p first calculates the ACF at varying distance lags, and then, the ACF is used to perform the slk correction where each *P* value is adjusted to adjacent *P* values as weighted according to the ACF. Any given *P* value will be pulled lower if its neighbors also have low *P* values and likely remain insignificant if the neighboring *P* values are also high. This is followed by a *q*-value score based on the Benjamini–Hochberg false discovery rate (FDR) correction. A peak-finding algorithm was used to find enrichment regions, and a *P* value for each region was assigned using the Stouffer–Liptak correction. The FDR *q*-value is used to define the extent of the region, whereas the slk-corrected *P* value and a one-step Sidak multiple-testing correction are used to define the significance of the region [33]. The parameters for Comb-p were DIST = 300, STEP = 60 and THRESHOLD = 0.05 [34]. We used GREAT to annotate DMCs/DMRs at a 50 kb proximity of the gene TSS [35]. The EnrichR package [36] was used to study the functional enrichment of biological processes of the genes associated with DMCs (at nominal *p* < 0.05) and DMRs (corrected *p* < 0.05). EnrichR uses the Fisher exact test and a correction test that is the *z*-score of the deviation from the expected rank by the Fisher exact test [36]. Gene network interactions were determined with GeneMANIA [37] and represented in the Cytoscape [38] platform.

Anthropometric data statistical analysis was performed using SPSS version 25.0 (SPSS Chicago, Illinois). Data are expressed as the mean and 95% confidence intervals (95% CI).

The Shapiro–Wilk test was used to determine whether the variables under study were normally distributed. To compare quantitative variables among the two groups included in the study, we used a *T* test for normally distributed variables. The relationships between categorical variables were evaluated by the *X*^2^ test. If the expected frequency of numbers less than 5 exceeded 20% of the calls, we used Fisher's exact test. *p* < 0.05 was considered statistically significant.

## Results

### Anthropometric data

A total of 25 newborns were included in the study: 13 LGA and 12 AGA. There were no significant group differences in terms of sex, type of delivery or gestational age (Table 1). There were significant differences in all anthropometric variables analyzed. At birth, LGA newborns were significantly heavier (4.32 vs. 3.24 kg, *P* < 0.001), longer (53.04 vs. 49.29 cm, *P* < 0.001) and had a larger head circumference (36.39 vs. 34.63 cm) than AGA newborns (Table 1).

**Table 1:** Anthropometric and demographic data of newborns

	AGA (n = 12)	LGA (n = 13)	*p* value
Gender: female/male (%/n)	50% (6)/50% (6)	46.2% (6)/53.8% (7)	*χ*^2^ NS
Type of delivery: vaginal/cesarean (%/n)	50% (6)/50% (6)	53.8% (7)/46.2% (6)	*Fisher NS*
Gestational age (weeks)	39.21 (38.49–39.93)	39.48 (38.72–40.25)	*t Student NS*
Birth weight (kg)	3.2 (2.9–3.5)	4.3 (4.2–4.5)	t Student***
Birth weight (SDS or Z-score)	0.03 (-0.46–0.53)	2.68 (2.24–3.12)	t Student***
Birth length (cm)	49.2 (48.3–50.2)	53.0 (52.1–53.9)	t Student***
Birth length (SDS)	-0.23 (-0.65–0.19)	1.92 (1.30–2.55)	t Student***
Birth ponderal index	2.7 (2.52–2.88)	2.9 (2.75–3.05)	*t Student *NS
Head circumference (cm)	34.6 (33.8–35.3)	36.3 (35.7–37.0)	t Student*
Head circumference (SDS)	0.04 (-0.42–0.51)	1.24 (0.84–1.63)	t Student***

*χ*^2^: Chi-square test * < 0.05; *** < 0.001; NS: not statistically significant

Due to the reduced sample size, we grouped maternal race into White and not White. In the AGA group, 4 newborns were not White (3 Hispanic and 1 Asian). In the LGA group, 6 newborns were not White (4 Hispanic and 2 Asians). No significant difference in maternal age at delivery or maternal race among the LGA and AGA groups was identified. We also did not observe differences between the two groups in the prevalence of maternal pregestational hypertension and diabetes, tobacco consumption during pregnancy or maternal glucose metabolism disturbances during pregnancy (Table 2). BMI at the beginning of pregnancy was not significantly different. However, the BMI increase during pregnancy was significantly higher in the LGA group (6.14 vs 4.27, *P* < 0.05) than in the AGA group. In addition, we observed nearly significant differences in maternal weight gain during pregnancy (Table 2).

**Table 2:** Maternal anthropometric, demographic and comorbidity data

	AGA (n = 12)	LGA (n = 13)	*p value*
Maternal age at the delivery (yr)	31.67 (27.55–35.78)	32.85 (28.91–36.78)	*t Student* NS
Maternal Race: white/not white (%/n)	66.7% (8)/33.3% (4)	53.8 (7)/46.2 (6)	*χ*^*2*^ NS
Maternal pregestational hypertension (%)	0	0	*Fisher* NS
Maternal pregestational diabetes (%/n)	0	15.38% (2)	*Fisher* NS
Maternal hypertension during pregnancy (%/n)	8.33% (1)	15.38% (2)	*Fisher* NS
Maternal glucose metabolism disturbances during pregnancy (%/n)	8.33% (1)	15.38% (2)	*Fisher* NS
Maternal glucose metabolism disturbances previous or during pregnancy (%/n)	8.3% (1)	30.8% (4)	*Fisher* NS
Smoking during pregnancy: yes/no (%)	0	0	*Fisher* NS
Maternal BMI at the start of pregnancy (kg/m^2^)	23.66 (21.20–26.10)	25.97 (23.58–28.35)	*t Student* NS
BMI increase during pregnancy	4.27 (3.40–5.14)	6.14 (4.74–7.55)	*t Student* *
% of maternal weight gain during pregnancy	18.6 (14.1–23.3)	24.4 (17.5–31.7)	*t Student* NS

*χ*^2^ = Chi-square test. * < 0.05; NS: not statistically significant

### DNA methylation data

#### Differentially methylated CpGs

**Fig. 1:**
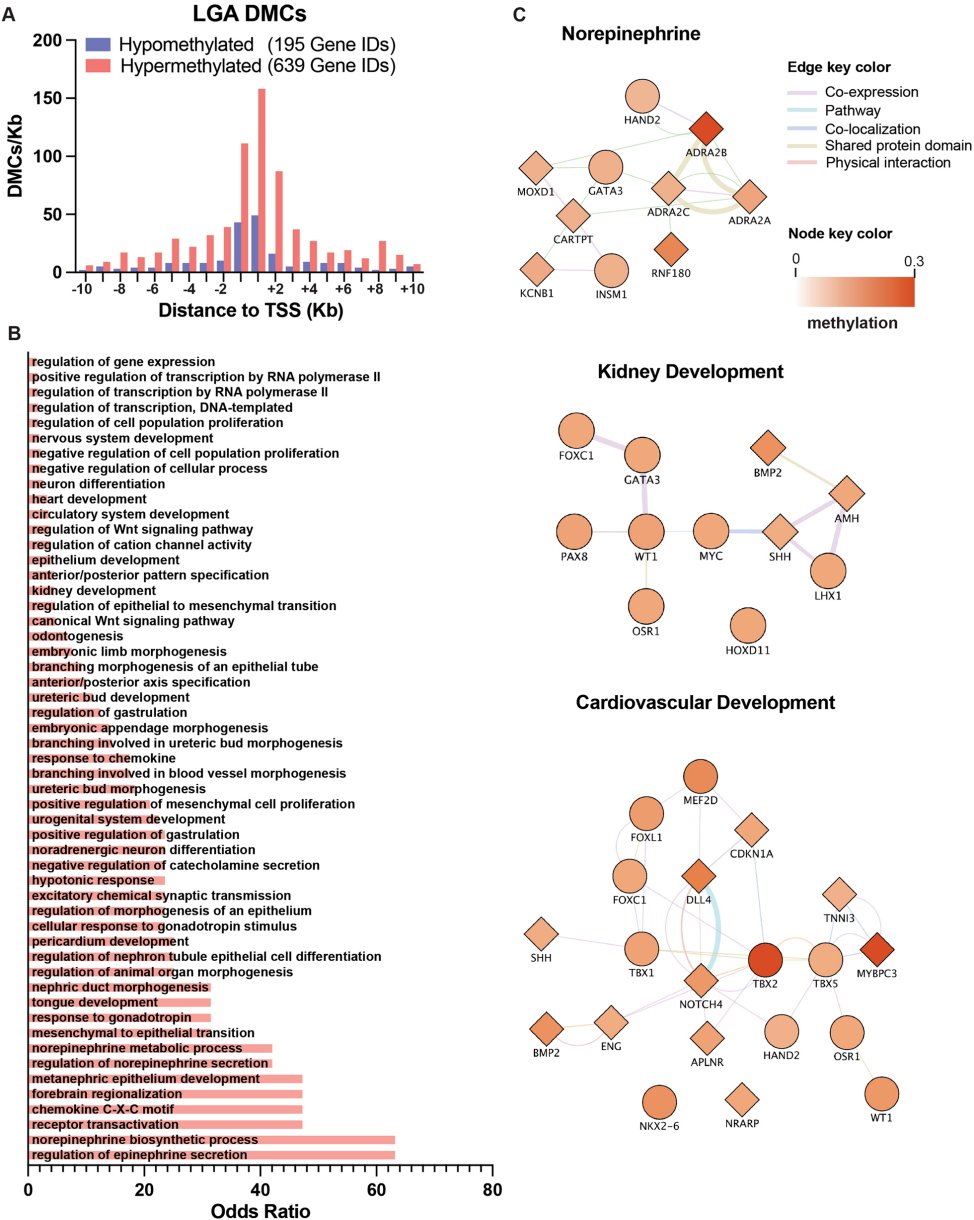
Identification of DMCs in LGA newborns. (**A**) Distribution of DMCs represented as the distance to the closest TSS, shown in 500 bp bins. Red bars denote the number of hypermethylated DMCs per bin, and blue bars denote hypomethylated DMCs per bin. (**B**) Enrichment analysis of the Gene Ontology (GO) category Biological Process (BP) of all hypermethylated DMCs with nominal *p* < 0.05. Only BPs with corrected *p* < 0.05 are shown. (**C**) Gene networks of selected BPs: Norepinephrine, Kidney Development and Cardiovascular Development. The methylation difference between LGA and AGA is shown as a graded color scale, where white is no change and red is hypermethylation. Genes (nodes) are shown either as circles or diamonds, where circles are those showing transcriptional activity according to GO regulation of transcription, DNA-templated (GO:0006355). Edges (connections/lines between nodes) represent co-expression, pathways, colocalization, shared protein domains or physical interactions between the two genes/proteins in the network according to GeneMANIA

After adjusting for covariables (gestational age and maternal weight gain during gestation), our analysis identified 1672 differentially methylated CpGs (DMCs) between the LGA and AGA groups with a nominal *p* < 0.05 (only two CpGs showed FDR < 0.05, see Additional file 6: Table 1). The distribution of these DMCs was 11 at 5 K promoter regions, 23 at the 3’ UTR regions, 15 at CpG islands, 141 at CpG shelves, 77 at CpG shores, and 703 at gene bodies with 48 at exons and 655 introns, while the remaining 969 CpGs were located in intergenic regions (Additional file 6: Table 1). Next, we identified 639 hypermethylated and 195 hypomethylated DMCs at 10 kb or less of any transcriptional start site (TSS) (Fig. 1A). Most of the DMCs were found in proximity to the gene’s TSS. Gene ontology (GO) analysis of these gene IDs identified several significantly enriched biological processes (BP) (adjusted *p* < 0.05) in the group of 639 hypermethylated DMCs (Fig. 1B), including regulation of transcription (GO:0006355), regulation of epinephrine secretion (GO:0014060), norepinephrine biosynthesis (GO:0042421), receptor transactivation (GO:0035624), forebrain regionalization (GO:0021871) and several terms related to kidney (GO:0060675, GO:0001658, GO:0001655, GO:0072182, GO:0001822, etc.) and cardiovascular development (GO:0001569, GO:0007507, GO:0060039, GO:0072359). No significant enrichment in gene ontology groups was found in the 195 gene IDs associated with hypomethylated DMCs.

The most significantly enriched GO group was Regulation of Transcription, with 114 hits out of 2244 (odds ratio = 1.33, adjusted *p* = 8.3E-5). Cytoscape representation of GeneMANIA gene networks for biological processes such as norepinephrine function, kidney development and cardiovascular development shows highly interconnected genes with hypermethylated DMCs in the LGA group (Fig. 1C). The Gene Ontology term Regulation of epinephrine secretion (GO:0014060) (odds ratio = 63.3, adjusted *p* = 0.0044) shows a cluster of 3 adrenergic receptors (*ADRA2A*, *B* and *C*) hypermethylated in the LGA group. Moreover, this cluster of adrenergic receptor genes is associated with two transcription factors, heart-and neural crest derivative-expressed 2 (*HAND2)* and GATA-binding protein 3 (*GATA3*). Hypermethylated DMCs were also enriched in a group of genes involved in kidney development, including several gene ontology terms, such as branching morphogenesis of an epithelial tube (GO:0048754) (odds ratio = 133, adjusted *p* = 6.88 × 10^–7^), ureteric bud morphogenesis (GO:0060675) (odds ratio = 18.5, adjusted *p* = 9.27 × 10^–7^), branching involved in ureteric bud morphogenesis (GO:0001658) (odds ratio = 161, adjusted *p* = 0.005), and urogenital system development (GO:0001655) (odds ratio = 247, adjusted *p* = 0.005). (Fig. 1B, Additional file 7: Table 2). Hypermethylated DMCs are associated with the Sonic Hedgehog (*SHH)* gene, which is involved in the establishment of cell fates during embryonic development [39]. Furthermore, a series of transcription factors involved in kidney development were also found to be associated with hypermethylated DMCs in LGA, including WT1 Transcription Factor (*WT1)*, Odd-Skipped-Related Transcription Factor 1 (*OSR1*), Lim Homeobox Gene 1 (*LHX1)* and MYC Protooncogene (*MYC)*.

Finally, a series of hypermethylated DMCs were found to be associated with genes involved in cardiovascular development (Fig. 1C), including several gene ontology terms, such as heart development (GO:0007507) (odds ratio = 34.9, adjusted *p* = 0.008), pericardium development (GO:0060039) (odds ratio = 233, adjusted *p* = 0.01), and circulatory system development (GO:0072359) (odds ratio = 32.6, adjusted *p* = 0.01) (Additional file 7 Table 2). Hypermethylated DMCs were associated with Delta-Like Canonical Notch Ligand 4 (*DLL4)* and Notch Receptor 4 (*NOTCH4)*, two genes involved in embryonic vascular development, vasculogenesis and angiogenesis, arterial and venous identities and the regulation of vessel branching [40]. The family of T-Box transcription factors (*TBX1, TBX2* and *TBX5)* and Myosin-Binding Protein C (*MYBPC3)* are involved in the development of the pharyngeal arch arteries [41], formation of the chambers of the myocardium and cardiomyocyte development [42].

To further validate our GO results, we used two additional systems biology approaches: analysis of canonical pathways using WikiPathways [36, 43–45] and the enrichment of genes associated with rare diseases [46]. This approach serves to further support our GO results by comparing our dataset with curated knowledge-based platforms that inform proteomic and metabolomic pathways [43] as well as pathological processes linked to single gene mutations [46]. This approach is especially helpful when working with small groups where significance by multiple testing correction is not met. Finding commonalities between outputs from different databases supports the overall findings of our study. Some of the top pathways (WikiPathways) enriched in hypermethylated DMCs were involved in heart development (WP1591: odds ratio 7.01; adjusted *p* = 0.015), development of the ureteric collection system (WP5053: odds ratio 6.52; adjusted *p* = 0.015) and lncRNA involved in canonical WNT signaling and colorectal cancer (WP4258: odds ratio 4.22; adjusted *p* = 0.018) (Table 3).

**Table 3:** Pathways significantly enriched in hypermethylated DMCs in the LGA group according to WikiPathways [36]

Term	Overlap	*P* value	Adjusted *P* value	Odds ratio	Combined score	Genes
Heart Development WP1591	8/44	5.15E-05	0.015	7.071	69.826	*TBX1;FOXC1;SHH;BMP2;HAND2;FOXH1;TBX5;TBX2*
Development of ureteric collection system WP5053	8/47	8.42E-05	0.015	6.526	61.233	*FOXC1;SHH;WT1;SIX2;LHX1;GATA3;FAT4;HOXD11*
LncRNA involvement in canonical Wnt signaling and colorectal cancer WP4258	11/94	1.47E-04	0.018	4.228	37.300	*APC2;TFAP2A;WNT10A;WNT2B;MYC;FZD7;TCF7;DVL1;FZD10;NOTUM;NKD1*
Breast cancer pathway WP4262	14/154	3.00E-04	0.022	3.196	25.934	*APC2;WNT10A;CDKN1A;WNT2B;NOTCH4;FZD7;TCF7;FZD10;FGF17;DLL4;MYC;DVL1;ERBB2;PGR*
ncRNAs involved in Wnt signaling in hepatocellular carcinoma WP4336	10/86	3.08E-04	0.022	4.192	33.893	*WNT10A;WNT2B;MYC;FZD7;TCF7;DVL1;FZD10;NOTUM;KLF4;NKD1*
Somatic sex determination WP4814	4/14	6.97E-04	0.043	12.662	92.038	*NR5A1;WT1;AMH;PTGDS*
Melatonin metabolism and effects WP3298	6/37	8.58E-04	0.045	6.140	43.356	*ACHE;MTNR1A;AANAT;CYP1B1;APOE;FOXO1*

Only pathways with corrected *p* < 0.05 are shown

Moreover, hypermethylated DMCs were found to be significantly associated with several rare diseases [46], most of which can be clustered into 4 groups: 1—skeletal defects (brachial arch defects, ulnar-mammary syndrome, dominant cleft palate, symphalangism distal, split hand and foot distal, Gordon syndrome, cleft lip and/or palate with mucous cysts of lower and Talipes equinovarus); 2—renal defects (renal agenesis, Mayer-Rokitansky–Kuster–Hauser syndrome); 3—cardiovascular (aortic arch defect); and 4—cancer (malignant cylindroma, urethral cancer, glass cell carcinoma of the cervix, testicular cancer). Most of these diseases are characterized by cardiac, renal, reproductive, and skeletal malformations (Table 4) and are associated with alterations in growth trajectories. This systems biology approach is used to identify genes affected by DMCs in LGA newborns that are enriched in disease-related pathways, giving further confidence in DMC identification, especially when using a small number of samples and an uncorrected analysis.

**Table 4:** Rare diseases significantly enriched in hypermethylated DMCs in the LGA group according to Enrichr [36]

Term	Overlap	*P* value	Adjusted *P* value	Odds ratio	Combined score	Genes
Branchial arch defects	17/142	1.83E-06	0.004	4.372	57.765	*GCM2;TFAP2A;TBX1;FOXC1;TWIST2;GATA3;KY;TBX2;SHH;CYP26B1;CXCL12;SIX2;HAND2;PAX9;MSX1;PITX1;NKX2-3*
Ulnar-mammary syndrome	11/63	3.11E-06	0.004	6.759	85.712	*TFAP2A;TBX1;FGF17;TBX15;CDKN1A;SHH;BMP2;MYC;TBX5;JUNB;TBX2*
Dominant cleft palate	23/267	9.61E-06	0.009	3.042	35.149	*TFAP2A;TBX1;CDKN1A;KCNK9;WNK4;TWIST2;HOXC13;KLF4;KY;CKAP4;SKI;SHH;BMP2;TERT;PTER;ESRP2;MYC;PAX9;KRT14;DVL1;COL9A3;MSX1;PITX1*
Renal agenesis bilateral	11/77	2.29E-05	0.016	5.321	56.867	*CDKN1A;PTER;PAX8;WT1;SIX2;LHX1;GATA3;NOTUM;AQP2;ACTB;MNX1*
Malignant cylindroma	22/282	6.75E-05	0.032	2.724	26.160	*FOXA1;CDKN1A;FOXC1;DYSF;GATA3;KLK8;KLK10;CKAP4;PURA;GJA1;CXCL12;TERT;PAX8;WT1;ALPP;MYC;ERBB2;CD9;COL9A3;PGR;CSK;ADA*
Mayer-Rokitansky-Kuster-Hauser syndrome	12/103	7.78E-05	0.032	4.212	39.852	*NR5A1;IL32;MUC1;SRD5A2;PTER;WT1;LHX1;WNT9B;PGR;AMH;TBX5;KY*
Urethral cancer	12/103	7.78E-05	0.032	4.212	39.852	*SHH;TERT;PAX8;ALPP;WT1;MYC;ERBB2;GATA3;AMH;KY;CKAP4;ADRA2A*
Symphalangism distal	11/94	1.47E-04	0.035	4.228	37.300	*SHH;HBM;BMP2;PTER;MYC;PRRX2;SP6;HOXC13;MSX1;GDF5;CKAP4*
Glassy cell carcinoma of the cervix	4/10	1.61E-04	0.035	21.108	184.317	*MUC1;ERBB2;KY;CKAP4*
Split hand foot malformation	14/146	1.72E-04	0.035	3.391	29.406	*WNT10A;AMN;LBX1;CKAP4;GJA1;SHH;PTER;MYC;LHX1;HAND2;PAX9;SP6;MSX1;ADA*
Testicular cancer	12/115	2.25E-04	0.035	3.719	31.244	*TFAP2A;CDKN1A;TERT;SRD5A2;PAX8;ALPP;WT1;MYC;ERBB2;CD9;AMH;KY*
22q11.2 deletion syndrome	12/116	2.44E-04	0.035	3.683	30.642	*GCM2;SLC25A1;TBX1;SHH;CYP26B1;PTER;HAND2;GATA3;PRODH;TBX5;MED15;TBX2*
Gordon syndrome	15/171	2.73E-04	0.035	3.076	25.246	*TBX1;IL11;OSR1;WNK4;STK39;AQP5;OXSR1;KY;SHH;PTER;PAX8;MYC;SCNN1B;WNK2;APOE*
Leydig cells hypoplasia	15/171	2.73E-04	0.035	3.076	25.246	*UCN;CDKN1A;EGR3;SRD5A2;OSR1;HSPA2;NR5A1;GJA1;BMP2;SHH;WT1;SIX2;ERBB2;PTGDS;AMH*
Meleda disease	11/101	2.80E-04	0.035	3.897	31.891	*UCN;GJA1;WNT10A;JUP;MYC;KRT14;AQP5;EVPL;KY;KRT6A;KRT9*
Palmoplantar keratoderma	11/101	2.80E-04	0.035	3.897	31.891	*UCN;GJA1;WNT10A;JUP;MYC;KRT14;AQP5;EVPL;KY;KRT6A;KRT9*
Cleft lip and/or palate with mucous cysts of lower	12/118	2.86E-04	0.035	3.613	29.483	*TFAP2A;TBX1;BMP2;SHH;TERT;PTER;BHMT2;PAX9;WNT9B;MSX1;KY;CKAP4*
Cleft lip palate-tetraphocomelia	12/118	2.86E-04	0.035	3.613	29.483	*TFAP2A;TBX1;BMP2;SHH;TERT;PTER;BHMT2;PAX9;WNT9B;MSX1;KY;CKAP4*
Aortic arches defect	5/20	2.88E-04	0.035	10.566	86.152	*TBX1;BMP2;HAND2;FOXH1;GATA3*
Talipes equinovarus	21/292	3.01E-04	0.035	2.489	20.180	*CDKN1A;OSR1;WNK4;STK39;HOXC13;HOXC12;HOXD11;GDF5;HOXD10;CELSR3;KY;TBX2;COL1A1;SKI;PURA;TERT;PTER;CYP1B1;HYLS1;MSX1;PITX1*

Only pathways with corrected *p* < 0.05 are shown

#### Differentially methylated regions (DMRs)

**Fig. 2:**
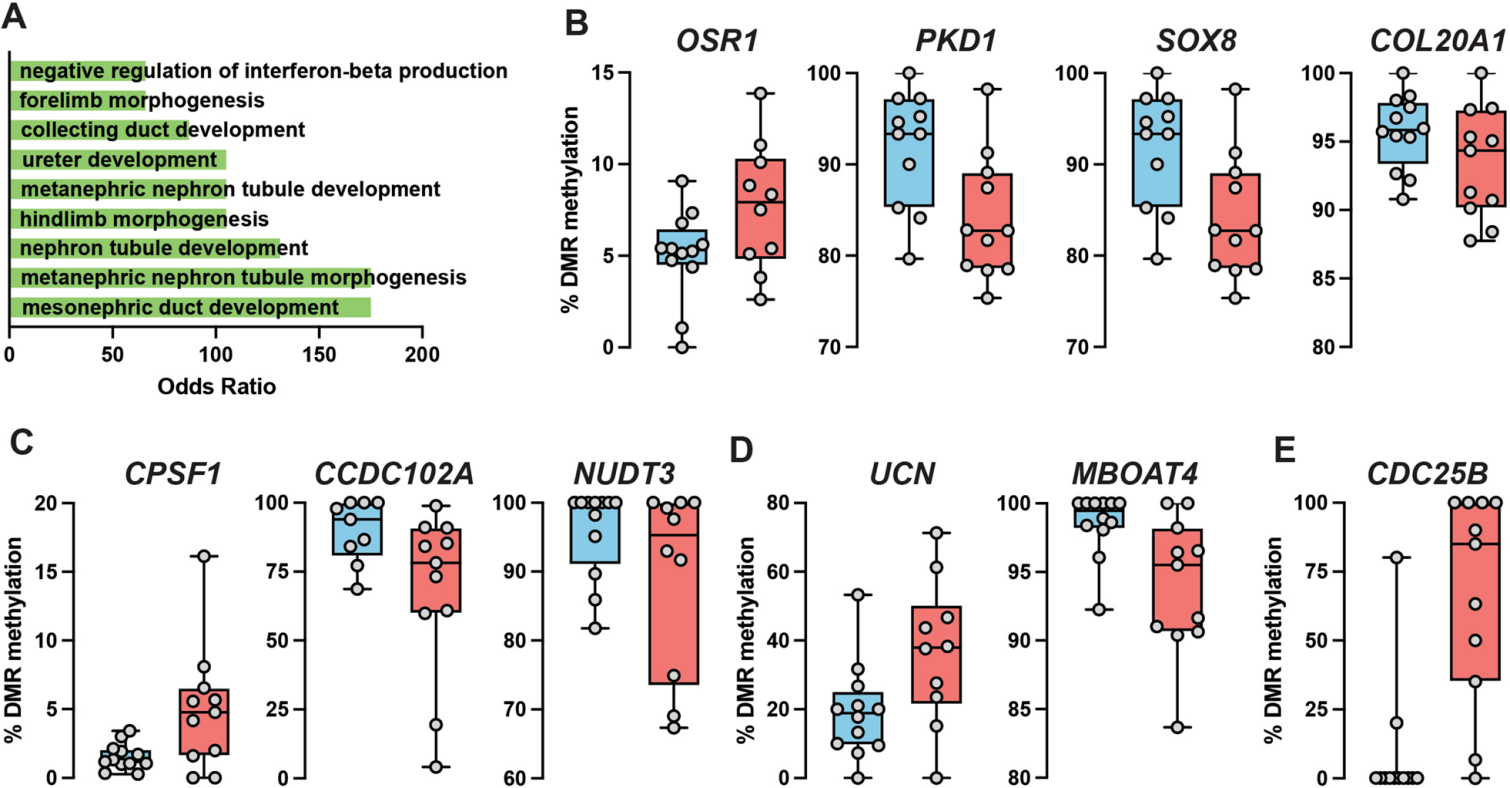
Identification of DMRs in LGA newborns. (**A**) Enrichment analysis of Gene Ontology (GO) category Biological Process (BP) of all DMRs with corrected *p* < 0.05 are shown. Percent methylation of DMRs associated with genes involved in kidney development (**B**), diabetic pathologies (**C**), metabolism and appetite (**D**) and cell division (**E**)

A total of 48 DMRs were identified between the LGA and AGA groups. The distribution of these DMRs was as follows: 9 at 5 K promoter regions, 4 at the 3’ UTR regions, 29 at CpG islands, 18 at CpG shelves, 10 at CpG shores, 38 at gene bodies with 20 at exons and 19 introns, while the remaining 10 CpGs were in intergenic regions (Additional file 8: Table 3). Gene ontology analysis of the genes in association with the 48 DMRs identified several significantly enriched biological processes related to kidney development, including Mesonephric duct development (GO:0072177) (odds ratio = 174, adjusted *p* = 0.03), Nephron tubule development (GO:0072080) (odds ratio = 131, adjusted *p* = 0.03), Hindlimb morphogenesis (GO:0035137) (odds ratio = 104, adjusted *p* = 0.03), and Negative regulation of interferon-beta production (GO:0032688) (odds ratio = 65.5, adjusted *p* = 0.05) (Fig. 2A and Additional file 9: Table 4). DMRs associated with kidney development included a DMR hypermethylated in LGA patients (Fig. 2B), composed of 7 CpGs located in the intron 1 region of the *OSR1* gene (Additional file 8: Table 3 and Additional file 1: Fig. 1), and 3 DMRs hypomethylated in LGA patients (Fig. 2B), composed of 3 CpGs located in the intron 1 region of the Polycystin 1 (*PKD1*) gene, 15 CpGs located in a CpG island in proximity of the SRY-Box 8 (*SOX8*) gene and 10 CpGs located in promoter region of the Collagen Type-20 Alpha-1 (*COL20A1*) gene (Additional file 8: Table 3).

A series of DMRs were found to be associated with genes linked to different diabetic pathologies. A DMR hypermethylated in LGA patients (Fig. 2C and Additional file 8: Table 3) was composed of 15 CpGs located in the promoter region of the cleavage and polyadenylation specific factor 1 (*CPSF1*) gene. Two DMRs were hypomethylated in LGA patients, one composed of 11 CpGs located in intron 1 of the coiled-coil domain containing 102A (*CCDC102A*) gene and the other composed of 7 CpGs located in the promoter region of the Nudix hydrolase (*NUDT3*) gene (Fig. 2C, Additional file 8: Table 3 and Additional file 2: Fig. 2).

Two DMRs were found in proximity of two genes involved in metabolism and control of appetite. One DMR, hypermethylated in LGA patients, is composed of 5 CpGs located in the intron 1—exon 2 boundary of the Urocortin (*UCN*) gene (Fig. 2D, Additional file 8: Table 3 and Additional file 3: Fig. 3). (DMR methylation rate: AGA = 19.2% vs LGA = 34.4%; *p* = 0.0001) (Additional file 8: Table 3). CpGs 2 and 3, located in the 5’ region of exon 2, are the most variable of the group (CpG2: AGA = 13.1% vs LGA = 29.7.4%; CpG3: AGA = 5.6% vs LGA = 40.5%).

The other DMR, hypomethylated in LGA patients, is composed of 4 CpGs and is in the promoter region of the Membrane-Bound O-Acetyltransferase Domain-Containing Protein 4 (*MBOAT4*) gene a.k.a. GOAT (for Ghrelin-O-Acyltransferase) (Fig. 2D, Additional file 8: Table 3 and Additional file 4: Fig. 4). The DMR at the *MBOAT4* locus is located at the promoter region (− 1473 to − 1515 bp from the TSS) (DMR methylation rate: AGA = 98.5% vs LGA = 94%; *p* = 4.6 × 10^–6^) (Additional file 8: Table 3). CpGs 2 and 3 were the most variable of the group (CpG2: AGA = 98.8% vs LGA = 91.7.4%; CpG3: AGA = 99.4% vs LGA = 93%) (Additional file 4: Fig. 4).

It is worth noting that the DMR with the highest methylation differences and hypermethylated in LGA patients is composed of 4 CpGs and located in intron 3 of the Cell Division Cycle 25B (*CDC25B*) gene (Fig. 2E and Additional file 8: Table 3). This gene regulates progression through the cell division cycle. Female *Cdc25b*-deficient mice are sterile due to permanent meiotic arrest of the oocyte [47].

**Fig. 3:**
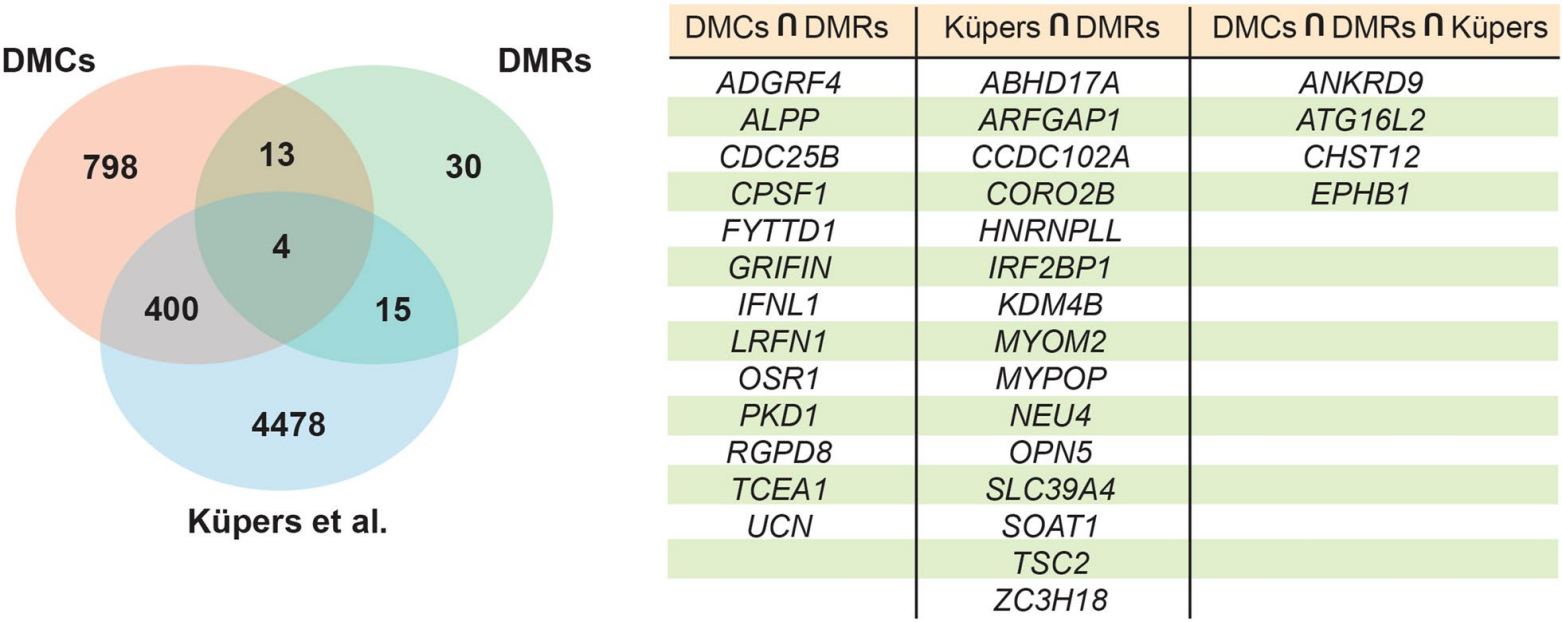
Identification of common Gene IDs identified by our DMC and DMR analysis in comparison with that of Küpers et al. [22], who used body weight at birth as a continuous variable. The Venn diagram shows the overlap between datasets with the numbers of Gene IDs in each. The table shows the intersection of gene IDs identified by our DMR and DMC analysis as well as DMR and genes from the Küpers et al. paper

To determine if our study showed overlap with previously published epigenome wide association studies (EWAS), we compared the Gene IDs identified by our DMCs (Additional file 6: Table 1) and DMRs (Additional file 8: Table 3) analysis with those identified by a meta-analysis of birthweight and DNA methylation at birth of 8,825 neonates by Küpers et al. [22]. The 48 DMRs identified here are near 62 genes, 32 out of 62 (approx. 52%) overlapped with either DMCs found by us (13 of 62: *ADGRF4, ALPP, CDC25B, CPSF1, FYTTD1, GRIFIN, IFNL1, LRFN1, OSR1, PKD1, RGPD8, TCEA1, UNC*), by Küpers et al. (15 of 62: *ABHD17A, ARFGAP1, CCDC102A, CORO2B, HNRNPLL, IRF2BP1, KDM4B, MYOM2, MYPOP, NEU4, OPN5, SLC39A4, SOAT1, TSC2, ZC3H18*) or both (4 of 62: *ANKRD9, ATG16L2, CHST12, EPHB1*) (Fig. 3).

## Discussion

### Main findings

The premise of our work was that there is an association between birthweight and DNA methylation in cord blood at birth. Thus, we sought to identify genome-wide methylation changes in normally occurring divergent growth trajectories by comparing methylation patterns from cord blood of LGA and AGA newborns. While LGA babies have a 1.5-fold increased risk of adult obesity [48], LGA babies are also associated with a higher risk of adult type 1 diabetes [49, 50] and a small but significant association with type 2 diabetes [51]. Our small cohort of 25 mother–infant pairs was extensively characterized to ensure that no major defects, malformations or syndromes were detected in the newborns and that no significant metabolic or cardiovascular deficiencies were identified in the mothers. We found 1672 DMCs and 48 differentially DMRs between the LGA and AGA groups. Due to the small sample size, we used nominal *p* values for DMC analysis, while we used multiple testing correction in DMRs and posterior system biology approaches. DMCs were significantly enriched with genes associated with the regulation of transcription, regulation of epinephrine secretion, norepinephrine biosynthesis, receptor transactivation, forebrain regionalization and several terms related to kidney and cardiovascular development. Furthermore, our dataset identified several DNA methylation markers enriched in gene networks involved in biological pathways and rare diseases of the cardiovascular system, kidneys, and metabolism. DMRs were found to be significantly enriched in processes related to kidney development, including mesonephric duct development and nephron tubule development. Approximately half of our DMR-associated genes overlapped with DMCs identified by us or by a previous epigenetic meta-analysis of body weight and DNA methylation at birth [22].

### Differential methylation in cardiovascular networks

The association between cardiovascular disease and birth weight is less well defined, while LGA is associated with increased risk hypertension during childhood and adolescence [52]; this relationship seems to be lost or even reversed in later life [51]. Some of these cardiovascular outcomes seem to be age- and/or sex-specific. LGA men but not women have a higher risk of poor cardiac autonomic function [53], while independent of gender, LGA adults were found to have an increased thickness of the radial artery and carotid artery intima [53, 54]. Overall, our DMC and DMR discovery pinpoints several heavily enriched biological processes involved in cardiovascular development and canonical pathways enriched with genes associated with a rare disease of the aortic arches [36, 43]. Hypermethylated DMCs were associated with *DLL4* and *NOTCH4*, two genes involved in embryonic vascular development, vasculogenesis and angiogenesis, arterial and venous identities and the regulation of vessel branching [40]. Additionally, several transcription factors involved in cardiovascular development and function were targeted by hypermethylated DMCs, such as *HAND2,* which plays a role in cardiac and aortic morphogenesis [55], *GATA3*, which is involved in endothelial cell biology and renal dysplasia when mutated [56], and T-Box transcription factors (*TBX1, TBX2* and *TBX5)* and myosin-binding protein C (*MYBPC3)*, which are involved in the development of the pharyngeal arch arteries [41], formation of the chambers of the myocardium and cardiomyocyte development [42]. Our analysis also identified a cluster of DMCs hypermethylated in proximity to alpha-2-adrenergic receptors (ADRA2) (A, B and C). These receptors regulate cardiovascular function when activated in the heart, blood vessels and kidney [57]. ADRA2A and ADRA2C are essential for the presynaptic control of neurotransmitter release, impacting plasmatic noradrenaline levels and ventricular contractility [58]. On the other hand, single nucleotide polymorphisms at the *ADRA2B* locus are associated with variations in the basal metabolic rate in obese populations [59] and adult metabolic disorders [60]. Today, our methylation data are the only association between LGA and ADRA2 function. These results may reveal an intimate relationship between alterations in prenatal growth trajectories and gene networks that control the development of the cardiovascular system.

### Differential methylation in renal networks

The present study identified several DMCs and DMRs enriched in loci involved in kidney development, morphogenesis and function. Gene ontology analysis identified several functions related to kidney development and function at the level of DMCs and DMRs. Hypermethylated DMCs were associated with the *SHH* gene involved in the establishment of cell fates during embryonic development [39]. A series of transcription factors (*WT1*, *OSR1, LHX1, MYC and SOX8)* involved in kidney development were also found to be associated with hypermethylated DMCs in LGA newborns. *WT1* is required for the normal formation of the genitourinary system [61]. Deletions of the *WT1* locus result in the formation of Wilms tumors, the most common renal tumor in children [62]. *OSR1 and LHX1* are key transcription factors involved in the regulation of nephron progenitor cells [63]. *MYC* is a master regulator of several genes involved in cell growth and cell cycle progression [64]. Deregulated *MYC* expression results in a variety of oncogenic processes as well as polycystic kidney disease [65]. *SOX8* is a transcription factor involved in the regulation of cell fate determination during embryonic development [66]. This transcriptional hub controls the normal development of the genitourinary system [61] by means of regulation of cell growth and cell cycle progression [64] as well as regulation of the nephron progenitor cell [63] population. Furthermore, hypomethylated DMRs were found at the *PKD1* and *COL20A1* loci of LGA newborns. While *PKD1* is involved in the maintenance of renal epithelial differentiation and organization [67], single nucleotide polymorphisms in the *COL20A1* locus are associated with diabetic kidney disease [68]. Inactivating mutations of the *PKD1* gene are responsible for different forms of autosomal dominant polycystic kidney disease [67]. Genome Wide Association Studies (GWAS) identified a series of single nucleotide polymorphisms in *COL20A1* in association with diabetic kidney disease [68].

This was further validated by the enrichment of families of genes involved in rare diseases of the kidney, such as renal agenesis and Mayer-Rokitansky–Kuster–Hauser syndrome. Although there are numerous studies linking low birth weight with kidney mass, nephron number and early onset chronic kidney failure [69–72], there are currently no studies linking adult renal dysfunction in individuals born large for gestational age. Our study identified several pathways involved in kidney development that are targeted by differential methylation in patients with divergent growth trajectories. The association between some overgrowth syndromes and a predisposition to cancer is well known, such as Beckwith-Wiedemann syndrome (BWS), Simpson–Golabi–Behmel and segmental overgrowth PTEN hamartoma syndrome, among other syndromes [73]. In children with overgrowth disorders, such as BWS, birth weight correlates with the size, number, and proliferative potential of muscle stem cells [74]. BWS patients have a higher incidence of malignancies, including hepatoblastoma, neuroblastoma, rhabdomyosarcoma, adrenal carcinoma and, above all, Wilms tumors [75, 76]. Our DMR analysis combined with a systems biology approach identified an enrichment of differential DNA methylation patterns in gene networks involved in several malignant processes, including malignant cylindroma, urethral cancer, glioblastoma cell carcinoma of the cervix and testicular cancer. Most of these rare diseases are characterized by cardiac, renal, reproductive, and skeletal malformations. Moreover, we identified hypermethylated DMCs at the *WT1* locus, which are required for the normal formation of the genitourinary system [61] and responsible for the formation of Wilms tumors, a renal tumor in children [62]. Our dataset GO enrichment analysis shows and overlaps with partial phenotypes of specific overgrowth syndromes caused by single gene mutations, furthering a link between the prenatal environment, epigenetic alterations, and postnatal health outcomes. The use of a systems biology approach comparing our dataset with pathways and disease outcomes is intended to enhance the validity of our results, especially when there are some similarities in the pathophysiology of adult LGA and diseases with clear genetic/pathway alterations.

### Differential methylation in metabolic networks

A series of DMRs were found to be associated with genes linked to different diabetic pathologies, metabolism, and control of appetite. A DMR hypermethylated in the *CPSF1* gene is a mediator of retinal vascular dysfunction in diabetes mellitus [77]. Two DMRs hypomethylated in the *CCDC102A* and *NUDT3* genes. While genomic variations in the *CCDC102A locus* were found to be associated with diabetic cataract [78], polymorphisms at the *NUDT3* locus were associated with body mass index (BMI), adiposity and pediatric onset type 2 diabetes [79]. Moreover, we also identified two DMRs in genes involved in metabolism and the control of appetite. One DMR close to the *UCN* gene was hypermethylated in LGA patients. This gene is involved in the suppression of appetite under stress conditions and acts as a CRF-like factor in producing anxiety-like effects [80]. Lasting hypermethylation of this region could induce downregulation of UCN expression and a blunted response to its appetite suppressive activity, leading to sustained overfeeding, long-term body weight gain and obesity. On the other hand, LGA newborns had a hypomethylated DMR in the *MBOAT4* gene regulatory region. MBOAT4 is responsible for acylation of ghrelin at serine 3, making it physiologically active and stimulating appetite and hunger in the feeding centers of the brain through activation of its cognate receptor growth hormone secretagogue receptor type 1 (GHSR1A). *MBOAT4* is regulated by nutrient availability, linking dietary lipids to energy expenditure [81]. Long-term overexpression of *MBOAT4* could induce a blunted response to a lipid-rich diet [82].

Several gene IDs targeted by DMRs also overlapped with DMCs outside the DMR region, including those of UCN, PKD OSR1 and CDC25B. More importantly, 19 out of 63 Gene IDs targeted by DMRs were also identified by a meta-analysis of 24 EWAS in newborn blood in association with birthweight. It is not uncommon to see a small overlap between EWAS, especially when the study population, methods and bioinformatic approaches differ.

## Limitations

The pathophysiology of LGA is a complex and multifactorial phenomenon influenced by a combination of genetic, maternal–fetal environmental, and epigenetic factors. The relative contribution of these factors can vary from case to case, making it essential to consider all three factors in understanding macrosomia. Variations in genes related to insulin sensitivity, glucose metabolism, and growth hormone can influence fetal growth. In some cases, familial patterns of macrosomia can be observed, suggesting a strong genetic component. On the other hand, maternal nutrition, maternal obesity, gestational diabetes, and other metabolic conditions are strongly associated with macrosomia. To identify the influence of DNA methylation in two distinct growth trajectories from our LARGAN cohort, we tried to minimize the contribution of genetic and maternal–fetal environment by recruiting patients with no history of macrosomia and with normal maternal glycemia and body weight gain during pregnancy to diminish the possible contribution of maternal diabetes. Despite the many studies examining the genetic, environmental, and epigenetic mechanisms linking early life growth with adult disease, very few common targets have been identified, a testament to the multifactorial nature of the growth process. Here, we hypothesize that divergent intrauterine growth trajectories impact DNA methylation sites on those gene networks associated with adult health outcomes, especially cardiometabolic health. Our study cannot discriminate between methylation changes that respond to differential growth trajectories from methylation changes that induce differential growth trajectories. Whatever the case, our data show that differential early growth trajectories impact DNA methylation patterns other than by chance, affecting pathways enriched in genes involved in cardiometabolic and kidney development. Previous studies identified very few DNA methylation patterns at birth that persisted into childhood or adulthood. This exposes the tantalizing possibility that transient changes in DNA methylation patterns during early life could have profound impacts in organ development and function [22]. Follow-up studies comparing anthropometric and physiological data at different ages are warranted to identify correlations with methylation levels at birth.

One of the biggest limitations of our study is the small sample size, preventing us from detecting small variations in DNA methylation and identifying DMCs with significant corrected p values. A larger study including a larger number of patients per group and with increased sequencing depth would not only permit the corroboration of the current findings but also identify other loci not identified under the current conditions.

While the goal of EWAS is to identify epigenetic regions associated with specific phenotypes, it is tempting to try to interpret the dataset and speculate on the potential impact of the DMCs/DMRs in gene expression/gene network function in the target tissue. We recognize that the use of surrogate tissue (blood cells) to identify changes in DNA methylation of inaccessible target tissues in living patients is a drawback but is the only means of study we have to identify epigenomic biomarkers of growth. Recent studies identified subsets of DMCs that correlate between blood and brain [83] and blood and liver [84], but further studies need to be done to generate a map of those sites informative of a wider range of tissues and cell types. Although the population studied was composed of diverse ethnicities, they were largely of white European origin. Future studies including ethnicity as a variable of study will be needed to understand how conserved these epigenetic associations are.

## Conclusions

Our study identified several epigenetic regions differentially methylated in association with fetal overgrowth. The use of cord blood as a material in combination with the TruSeq EPIC enrichment platform for the identification of epigenetic biomarkers gives us the possibility to perform follow-up studies on the same patients as they enter childhood and puberty. These studies will not only help us understand how the epigenome responds to continuum postnatal growth but also link early alterations of the DNA methylome with later clinical markers of growth and metabolic fitness.

### Supplementary Information

**Additional file 1**. **Additional Figure 1**: Schematic representation of DMR associated with the OSR1 locus and % methylation level of each CpG. Black boxes are coding exons, white boxes are noncoding exons or UTRs, and red boxes are CpGs. Positions refer to the gene’s TSS (+1).

**Additional file 2**. **Additional Figure 2**: Schematic representation of DMR associated with the NUDT3 locus and % methylation level of each CpG. Black boxes are coding exons, white boxes are noncoding exons or UTRs, and red boxes are CpGs. Positions refer to the gene’s TSS (+1).

**Additional file 3**. **Additional Figure 3**: Schematic representation of DMR associated with the UCN locus and % methylation level of each CpG. Black boxes are coding exons, white boxes are noncoding exons or UTRs, and red boxes are CpGs. Positions refer to the gene’s TSS (+1).

**Additional file 4**. **Additional Figure 4**: Schematic representation of DMR associated with the MBOAT4 locus and % methylation level of each CpG. Black boxes are coding exons, white boxes are noncoding exons or UTRs, and red boxes are CpGs. Positions refer to the gene’s TSS (+1).

**Additional file 5**. **Additional Figure 5**: Schematic representation of DMR associated with the CDC25B locus and % methylation level of each CpG. Black boxes are coding exons, and red boxes are CpGs. Positions refer to the gene’s TSS (+1).

**Additional file 6**. **Additional Table 1**: Differentially methylated CpGs (DMCs) identified when comparing the AGA vs LGA groups.

**Additional file 7**. **Additional Table 2**: Enriched biological processes (BP) of genes in proximity to DMCs analyzed by ENRICH.

**Additional file 8**. **Additional Table 3**: Differentially methylated regions (DMRs) identified when comparing the AGA vs LGA groups.

**Additional file 9**. **Additional Table 4**: Enriched biological processes (BP) of genes in proximity to DMRs analyzed by ENRICH.

## Data Availability

All data relevant to the study can be found at the GEO repository with accession number GSE238155 or uploaded as supplementary information.
