# Supplementary figures and images for: Methylation analysis by targeted bisulfite sequencing in large for gestational age (LGA) newborns: the LARGAN cohort
Source: Clin Epigenetics. 2023 Dec 13;15:191. doi: 10.1186/s13148-023-01612-8 (PMC10717641; doi:10.1186/s13148-023-01612-8)

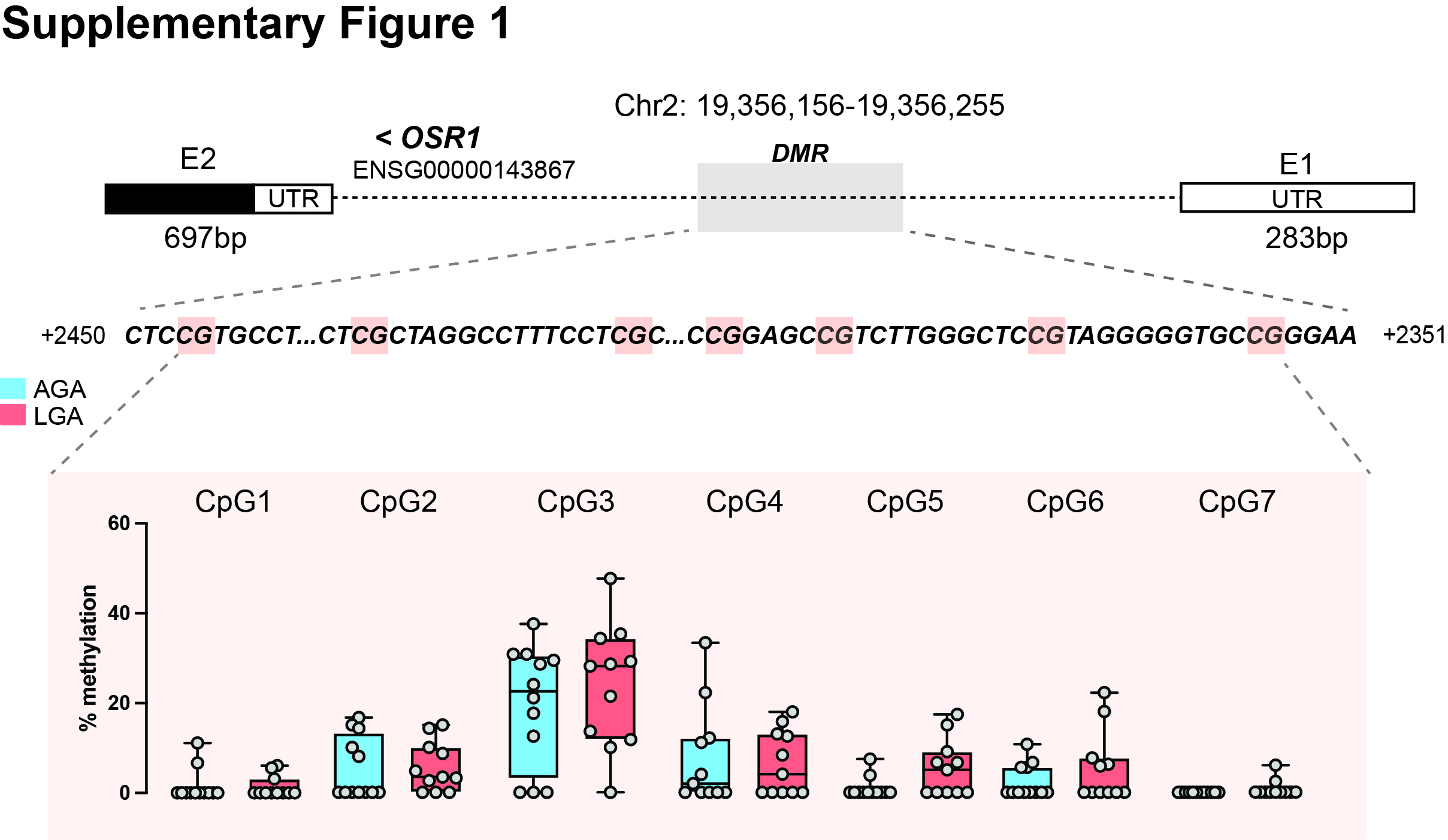

Supplement: Supplementary file 1 — Additional file 1. Additional Figure 1: Schematic representation of DMR associated with the OSR1 locus and % methylation level of each CpG. Black boxes are coding exons, white boxes are noncoding exons or UTRs, and red boxes are CpGs. Positions refer to the gene’s TSS (+1). [file 13148_2023_1612_MOESM1_ESM.jpg]

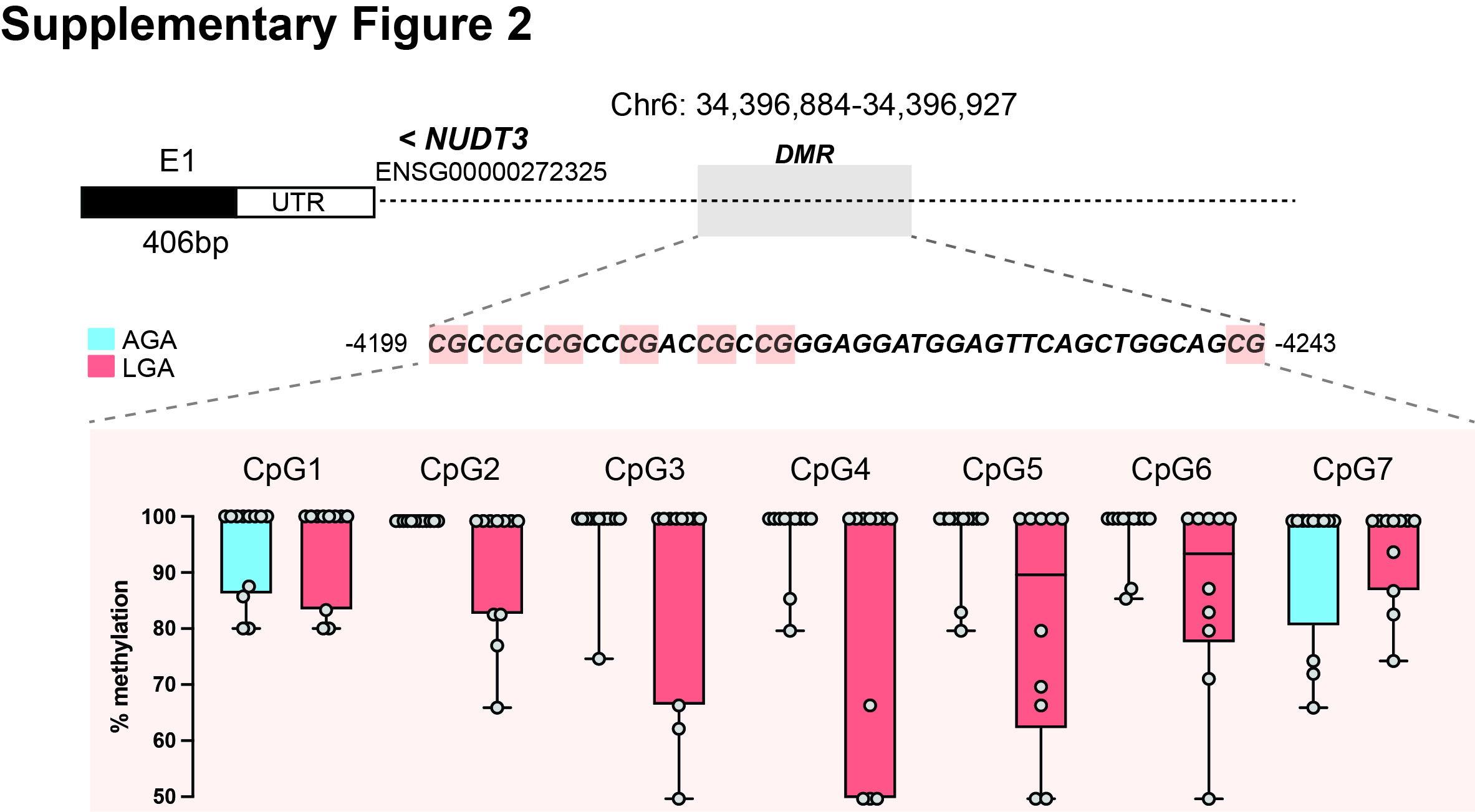

Supplement: Supplementary file 2 — Additional file 2. Additional Figure 2: Schematic representation of DMR associated with the NUDT3 locus and % methylation level of each CpG. Black boxes are coding exons, white boxes are noncoding exons or UTRs, and red boxes are CpGs. Positions refer to the gene’s TSS (+1). [file 13148_2023_1612_MOESM2_ESM.jpg]

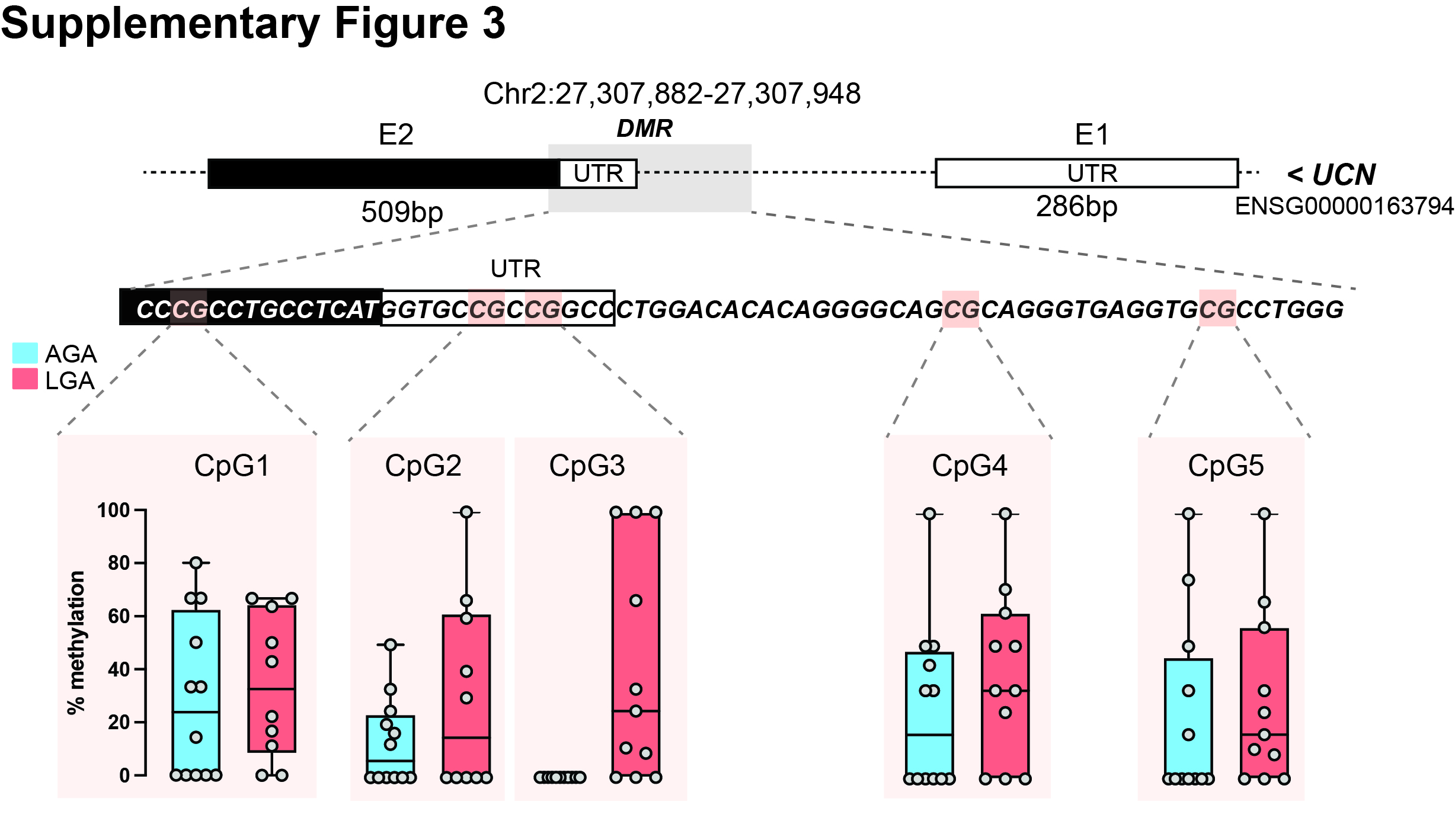

Supplement: Supplementary file 3 — Additional file 3. Additional Figure 3: Schematic representation of DMR associated with the UCN locus and % methylation level of each CpG. Black boxes are coding exons, white boxes are noncoding exons or UTRs, and red boxes are CpGs. Positions refer to the gene’s TSS (+1). [file 13148_2023_1612_MOESM3_ESM.jpg]

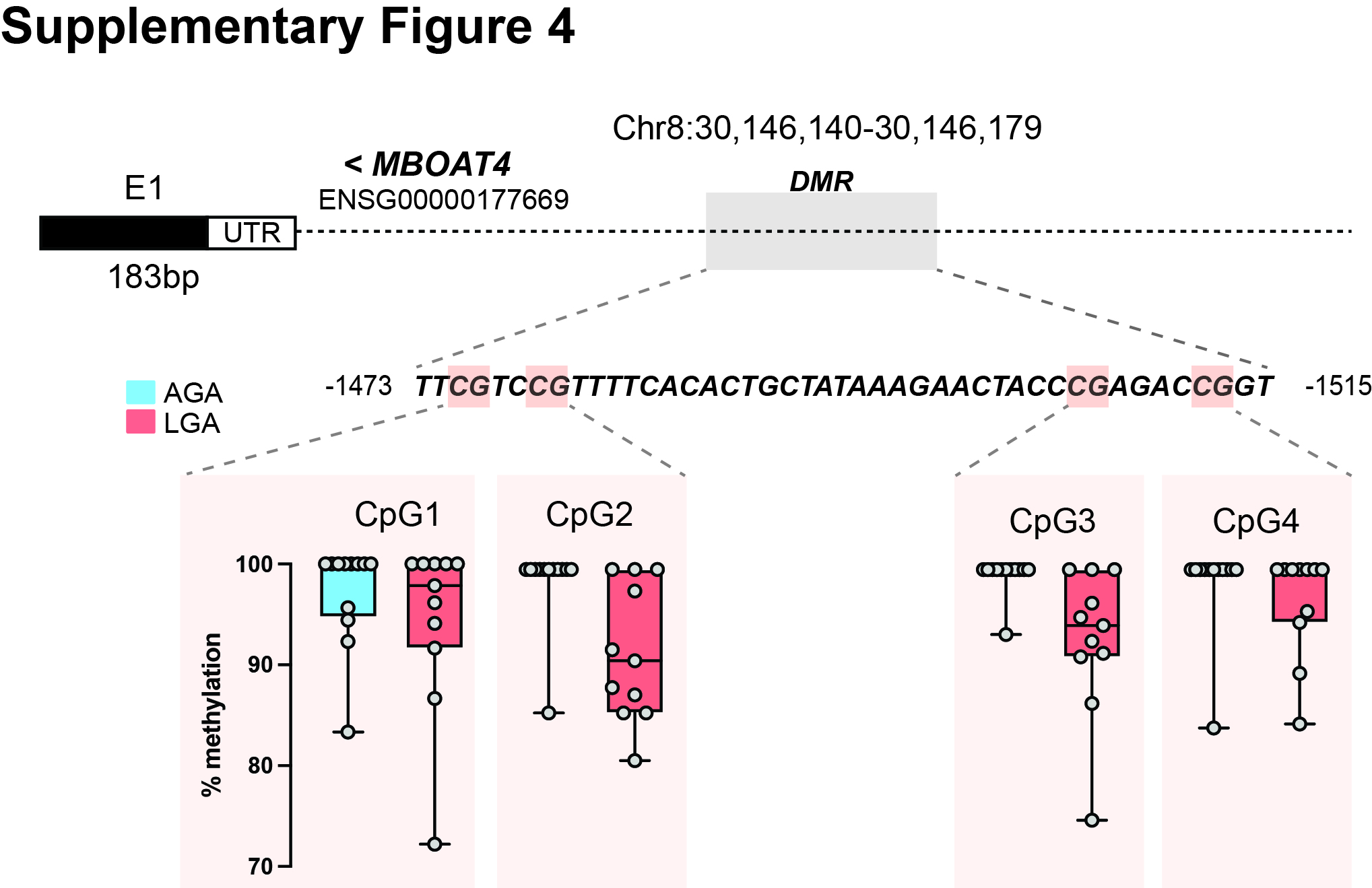

Supplement: Supplementary file 4 — Additional file 4. Additional Figure 4: Schematic representation of DMR associated with the MBOAT4 locus and % methylation level of each CpG. Black boxes are coding exons, white boxes are noncoding exons or UTRs, and red boxes are CpGs. Positions refer to the gene’s TSS (+1). [file 13148_2023_1612_MOESM4_ESM.jpg]

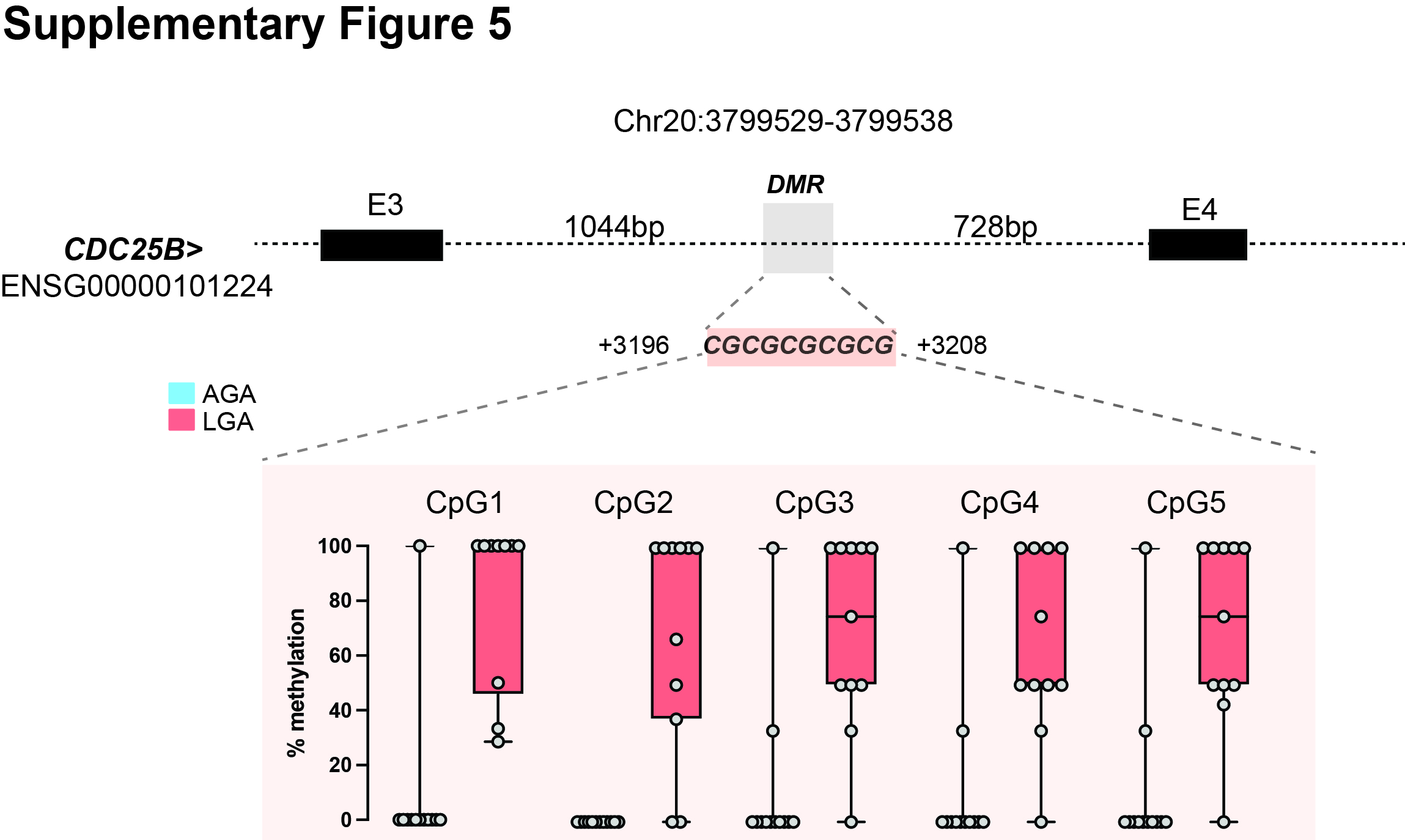

Supplement: Supplementary file 5 — Additional file 5. Additional Figure 5: Schematic representation of DMR associated with the CDC25B locus and % methylation level of each CpG. Black boxes are coding exons, and red boxes are CpGs. Positions refer to the gene’s TSS (+1). [file 13148_2023_1612_MOESM5_ESM.jpg]
